# Endoscopic Ultrasound‐Guided Gallbladder Drainage as a Bridge to Surgery for Acute Cholecystitis: A Comparative Study

**DOI:** 10.1002/deo2.70436

**Published:** 2026-09-29

**Authors:** Keiki Nagai, Yuji Fujita, Hiroki Uechi, Yuji Koyama, Shintaro Tsujikawa, Eisuke Suzuki, Seitaro Tsujino, Ken Ishii, Atsuki Nagao, Masato Yoneda

**Affiliations:** ^1^ Department of Hepato‐Biliary‐Pancreatic Medicine NTT Medical Center Tokyo Tokyo Japan; ^2^ Department of Hepato‐Biliary‐Pancreatic Surgery NTT Medical Center Tokyo Tokyo Japan; ^3^ Department of Gastroenterology and Hepatology Yokohama City University Hospital Kanagawa Japan

**Keywords:** acute cholecystitis, cholecystectomy, endoscopic transpapillary gallbladder drainage, endoscopic ultrasound‐guided gallbladder drainage, percutaneous transhepatic gallbladder drainage

## Abstract

**Objectives:**

Gallbladder drainage is used for patients with acute cholecystitis (AC) who are unsuitable for emergency cholecystectomy. Percutaneous transhepatic gallbladder drainage (PTGBD), endoscopic ultrasound‐guided gallbladder drainage (EUS‐GBD), and endoscopic transpapillary GBD (ETGBD) are used as bridge‐to‐surgery (BTS) approaches, but comparative data are limited. We compared clinical and perioperative outcomes among these strategies.

**Methods:**

This retrospective, single‐center study included 84 patients with AC who underwent GBD between January 2016 and January 2026 (PTGBD, *n* = 26; EUS‐GBD, *n* = 39; ETGBD, *n* = 19). The primary endpoint was technical success. Secondary endpoints included clinical success, adverse events, recurrent cholecystitis, hospital stay, and surgical outcomes after BTS.

**Results:**

Technical success rates were 100% in the PTGBD and EUS‐GBD groups and 73.7% in the ETGBD group (*p* < 0.001). Clinical success rates were 92.3%, 100%, and 85.7%, respectively. Recurrent cholecystitis occurred in 7.7% of PTGBD cases and 14.3% of ETGBD cases, but not in EUS‐GBD cases. Median hospital stay was the shortest in the EUS‐GBD group (7 vs. 13.5 days for PTGBD and 9 days for ETGBD; *p* < 0.001). Among patients who proceeded to surgery, laparoscopic cholecystectomy was performed in 100%, 87.5%, and 75.0% of the EUS‐GBD, PTGBD, and ETGBD groups, respectively (*p* = 0.041).

**Conclusions:**

All three approaches were feasible BTS strategies for AC. EUS‐GBD achieved high technical and clinical success with acceptable safety. After successful BTS, surgical outcomes were broadly comparable among groups. Drainage strategy should be individualized based on patient conditions, anatomical feasibility, and circumstances.

**Trial Registration**: N/A.

## 1 Introduction

Cholecystectomy is the definitive treatment for acute cholecystitis (AC), and the Tokyo Guidelines 2018 (TG18) recommend early laparoscopic cholecystectomy for surgically fit patients [1]. However, in older patients and those with severe comorbidities, emergency surgery may carry substantial perioperative risk. In such cases, gallbladder drainage (GBD) is often performed to control inflammation and sepsis and to allow delayed cholecystectomy under more favorable conditions.

Available drainage methods include percutaneous transhepatic GBD (PTGBD), endoscopic ultrasound‐guided GBD (EUS‐GBD), and endoscopic transpapillary GBD (ETGBD) [2, 3, 4]. PTGBD is the standard bridge‐to‐surgery (BTS) approach because of its wide availability and high technical success, but it requires an external tube and may be associated with discomfort, dislodgement, bleeding, bile leakage, and reintervention [2, 5, 6]. Among internal drainage approaches, EUS‐GBD has high technical and clinical success and does not depend on cystic duct patency [7], whereas ETGBD preserves native anatomy but may be technically limited by cystic duct stricture, inflammation, or impacted stones [8].

Although the efficacy and safety of these methods have been reported, comparative data on all three modalities in the BTS setting remain limited, particularly regarding subsequent surgical outcomes [9]. We previously compared EUS‐GBD and PTGBD at our institution [10], but a comprehensive evaluation including ETGBD has not been performed. Therefore, we compared the clinical outcomes of PTGBD, EUS‐GBD, and ETGBD as BTS strategies for AC, with emphasis on drainage and subsequent surgical outcomes.

## 2 Materials and Methods

### 2.1 Patients

We retrospectively identified consecutive patients from the electronic medical records and the institutional endoscopy procedure database of NTT Medical Center Tokyo. Between January 2016 and January 2026, 236 consecutive patients with acute cholecystitis who were unsuitable for emergency cholecystectomy and underwent GBD with the intention of subsequent cholecystectomy were identified. Patients with concomitant cholangitis due to common bile duct stones or suspected gallbladder perforation were excluded. Diagnosis and severity were assessed according to TG18 [1].

Of these 236 patients, 152 (PTGBD, *n *= 48; EUS‐GBD, *n* = 78; ETGBD, *n* = 26) did not subsequently undergo cholecystectomy and were excluded; the remaining 84 (PTGBD, *n *= 26; EUS‐GBD, *n *= 39; ETGBD, *n* = 19) constituted the analytic cohort for drainage outcomes (Figure 1).

**FIGURE 1:**
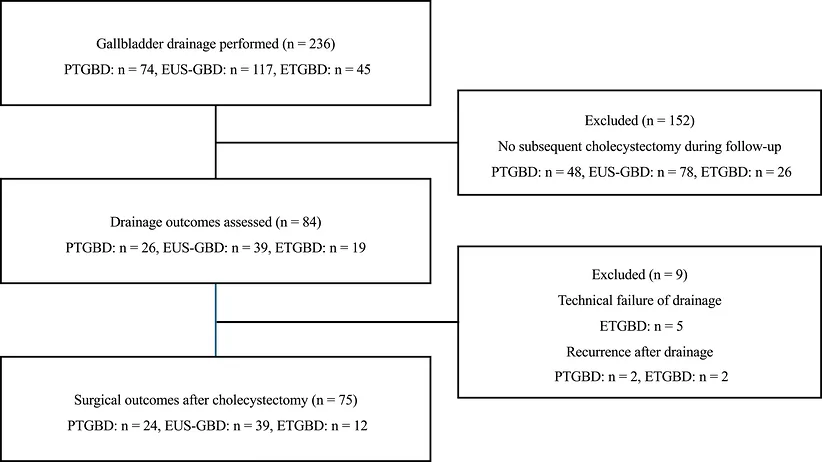
Patient flow diagram for the surgical outcome analysis after gallbladder drainage.

Patients were grouped according to the initial drainage method: PTGBD, EUS‐GBD, or ETGBD. The drainage method was selected by the treating physicians based on clinical condition, comorbidities, anatomical feasibility, and procedural considerations. The study was approved by the Institutional Review Board of NTT Medical Center Tokyo (Approval No. 200037461‐01) and was conducted in accordance with the Declaration of Helsinki. Written informed consent for study participation was waived, and an opt‐out approach was adopted for participant notification.

### 2.2 Procedures

#### 2.2.1 Percutaneous Transhepatic Gallbladder Drainage

PTGBD was performed under local anesthesia. The gallbladder was punctured via the transhepatic route, and bile aspiration and contrast injection were used to confirm gallbladder access. A guidewire was inserted, and a 7 Fr pigtail drainage catheter was placed under fluoroscopic guidance for external drainage.

#### 2.2.2 Endoscopic Ultrasound‐Guided GBD

EUS‐GBD was performed under fluoroscopic guidance using a linear echoendoscope (GF‐UCT260; Olympus Corp., Tokyo, Japan) with the patient in the prone position under intravenous sedation. In all cases, the endoscope was initially positioned in the duodenal bulb, where the gallbladder was visualized, and puncture was attempted from this position. Puncture was performed using a 19‐gauge needle (EZ Shot 3 Plus; Olympus Corp., Tokyo, Japan, or SonoTip TopGain; Medi‐Globe GmbH, Rohrdorf, Germany). After confirmation of bile reflux, contrast medium was injected to assess gallbladder position and morphology, and a 0.025 in guidewire (VisiGlide2; Olympus Corp.) was advanced into the gallbladder. The tract was dilated using a 7 Fr dilator (ES Dilator; Zeon Medical Inc., Tokyo, Japan) or a 4 mm balloon dilator (REN Balloon Catheter; Kaneka Corp., Osaka, Japan), followed by placement of a double‐pigtail plastic stent (DPPS; 7 Fr; 7, 10, or 15 cm; Through & Pass Double Pig Stent; Gadelius Medical Co., Ltd., Tokyo, Japan) for internal drainage. After stent placement, bile drainage and stent position were confirmed endoscopically or fluoroscopically, and the patient was assessed for bleeding or stent migration.

#### 2.2.3 Endoscopic Transpapillary Gallbladder Drainage

ETGBD was performed under fluoroscopic guidance using a side‐viewing duodenoscope (JF‐260 V, TJF‐260 V, or TJF‐Q290V; Olympus Corp.) with the patient in the prone position under intravenous sedation. After biliary cannulation and cholangiography to identify the cystic duct, a 0.025 in guidewire (VisiGlide2; Olympus Corp.) was advanced into the gallbladder, and internal drainage was established using either a 5 Fr plastic stent (Gadelius Medical Co., Ltd.) or a 7 Fr, 15 cm DPPS. Successful stent placement and bile drainage were confirmed fluoroscopically.

#### 2.2.4 Cholecystectomy

Cholecystectomy was performed laparoscopically or by an open approach. In the EUS‐GBD and ETGBD groups, internal stents were removed the day before surgery endoscopically; in the PTGBD group, surgery was performed after tube removal following tract maturation or with the tube left in place. Laparoscopic cholecystectomy was performed under general anesthesia using a four‐port technique, comprising adhesiolysis, fistula division with a linear stapler, achievement of the critical view of safety, division of the cystic duct and artery, and gallbladder dissection from the liver bed. When the critical view of safety could not be achieved, or the cystic duct was too thick for clipping, a fundus‐first approach and ENDOLOOP PDS II ligation (Johnson & Johnson, New Brunswick, NJ, USA) were used. Conversion to open cholecystectomy was performed when safe laparoscopic completion was not feasible.

### 2.3 Definitions

AC was diagnosed according to TG18 [1] based on local and systemic inflammatory signs and characteristic imaging findings. An attempted procedure was defined as a planned intervention initiated in the procedure room (PTGBD) or endoscopy suite (EUS‐GBD/ETGBD) after confirmation of the operator's indication, procedural informed consent, and intent to perform drainage. Interventions aborted before puncture or device deployment were classified as technical failures and retained in the technical‐success denominator. Technical success was defined as successful placement of a drainage device with confirmation of effective bile drainage. Clinical success was defined as improvement in abdominal symptoms and inflammatory markers within 72 h without additional intervention.

Procedure‐related adverse events were categorized according to the American Society for Gastrointestinal Endoscopy lexicon [11] and defined as events occurring between GBD and cholecystectomy. Recurrent cholecystitis was defined as recurrence of AC due to stent or catheter occlusion or dislodgement that required antibiotics or additional intervention.

For the surgical outcome analysis, patients with technically unsuccessful initial drainage requiring alternative drainage and those with recurrent AC requiring additional drainage or emergency surgery were excluded. Postoperative complications were evaluated using the Clavien–Dindo classification [12].

### 2.4 Endpoints

The primary endpoint was the technical success rate of GBD. Secondary endpoints included clinical success rate, procedure‐related adverse events, recurrent AC, the interval from drainage to cholecystectomy, and surgical outcomes following BTS. Surgical outcomes included the initial surgical approach, conversion to open surgery, operative time, estimated blood loss, postoperative complications, and length of postoperative hospital stay.

### 2.5 Statistical Analysis

Statistical analyses were performed using SPSS version 29 (IBM Corp., Armonk, NY, USA). Continuous variables are presented as median (interquartile range [IQR]) and categorical variables as number (percentage). Continuous and categorical variables were compared using the Kruskal–Wallis test and Fisher's exact test, respectively. Two‐sided *p*‐values <0.05 were considered statistically significant.

## 3 Results

### 3.1 Patient Characteristics

Figure 1 shows the patient flow, and Table 1 summarizes the baseline characteristics. Age, sex, etiology, history of upper gastrointestinal surgery, ASA‐PS, and TG18 severity grade did not differ significantly. Median body mass index differed significantly among the groups (*p* = 0.030), and cerebrovascular disease was more common in the ETGBD group (*p* = 0.045). No significant differences were observed in the other baseline comorbidities or use of antithrombotic therapy.

**TABLE 1:** Baseline characteristics.

Variable	PTGBD (*n* = 26)	EUS‐GBD (*n* = 39)	ETGBD (*n* = 19)	*p*‐Value
Age, years, median (IQR)	76.5 (66–84)	73 (58–81)	70.5 (60–79)	0.281
Male, *n* (%)	20 (76.9)	23 (59.0)	15 (78.9)	0.176
**Etiology, *n* (%)**				0.102
Gallstone	24 (92.3)	39 (100)	19 (100)	
Acalculous	1 (3.8)	0	0	
Gallbladder cancer	1 (3.8)	0	0	
History of upper gastrointestinal surgery, n (%)	3 (11.5)	2 (5.1)	0	0.259
BMI, kg/m^2^, median (IQR)	22.7 (20.0–25.6)	23.9 (21.5–27.8)	23.7 (22.2–27.6)	0.030
**ASA‐PS, *n* (%)**				0.403
I	1 (3.8)	5 (12.8)	3 (15.8)	
II	21 (80.8)	32 (82.1)	15 (78.9)	
≥III	4 (15.4)	2 (5.1)	1 (5.3)	
**Severity of cholecystitis according to TG18, *n* (%)**				0.587
Grade II	25 (96.2)	37 (94.9)	19 (100)	
Grade III	1 (3.8)	2 (5.1)	0	
**Comorbidities, *n* (%)**				
Hypertension	20 (76.9)	25 (64.1)	11 (57.9)	0.367
Diabetes mellitus	12 (46.2)	8 (20.5)	7 (36.8)	0.084
Dyslipidemia	11 (42.3)	12 (30.8)	9 (47.4)	0.411
Cardiac disease	10 (38.5)	6 (15.4)	6 (31.6)	0.097
Cerebrovascular disease	3 (11.5)	3 (7.7)	6 (31.6)	0.045
Chronic kidney disease	3 (11.5)	2 (5.1)	5 (26.3)	0.065
Hemodialysis	3 (11.5)	1 (2.6)	0	0.135
Chronic respiratory disease	6 (23.1)	2 (5.1)	2 (10.5)	0.089
Antithrombotic therapy, *n* (%)	10 (38.5)	7 (17.9)	7 (36.8)	0.133

*Note*: Data are presented as median (IQR) or *n* (%).

Abbreviations: ASA‐PS, American Society of Anesthesiologists physical status; BMI, body mass index; ETGBD, endoscopic transpapillary gallbladder drainage; EUS‐GBD, endoscopic ultrasound‐guided gallbladder drainage; IQR, interquartile range; PTGBD, percutaneous transhepatic gallbladder drainage; TG18, Tokyo Guidelines 2018.

### 3.2 Outcomes of GBD

Table 2 summarizes the drainage outcomes. Procedure time differed significantly among the groups (*p* < 0.001) and was the shortest in the PTGBD group and longest in the ETGBD group. Technical success was achieved in all patients in the PTGBD and EUS‐GBD groups but was lower in the ETGBD group (73.7%; *p* < 0.001). Clinical success and overall adverse event rates did not differ significantly. Recurrent cholecystitis occurred in the PTGBD and ETGBD groups but not in the EUS‐GBD group (*p* = 0.061). Median length of hospital stay also differed significantly (*p* < 0.001) and was the shortest in the EUS‐GBD group and longest in the PTGBD group. Drain dislodgement occurred only in the PTGBD group, peritonitis only in the EUS‐GBD group, and post‐ERCP pancreatitis only in the ETGBD group; all cases of peritonitis and post‐ERCP pancreatitis were mild and resolved with conservative management. All 39 EUS‐GBD procedures were performed using a Through & Pass Double Pig Stent (7 Fr; Gadelius Medical Co., Ltd., Tokyo, Japan). Although the gallbladder was visualized from the duodenal bulb in all cases, slight scope displacement during adjustment of the puncture site resulted in some punctures being performed just proximal to the pylorus from the gastric antrum. Thus, the gallbladder was accessed using a transgastric approach in nine patients (23.1%) and a transduodenal approach in 30 patients (76.9%). A 10 cm stent was used in 37 patients (94.9%), whereas 7 and 15 cm stents were each used in one patient (2.6%); these two patients underwent transduodenal EUS‐GBD.

**TABLE 2:** Outcomes of gallbladder drainage.

Variable	PTGBD (*n* = 26)	EUS‐GBD (*n* = 39)	ETGBD (*n* = 19)	*p*‐Value
Procedure time, min, median (IQR)	16.5 (13.3–21.5)	21 (17–28)	38.0 (21.5–51.5)	<0.001
Technical success, *n* (%)	26 (100)	39 (100)	14 (73.7)	<0.001
Clinical success, *n* (%)	24 (92.3)	39 (100)	12 (85.7)	0.082
Adverse events, *n* (%)	5 (19.2)	6 (15.4)	2 (10.5)	0.561
Drain dislodgement	5 (19.2)	0	0	
Peritonitis	0	6 (15.4)	0	
Post‐ERCP pancreatitis	NA	NA	2 (10.5)	
Recurrent cholecystitis	2 (7.7)	0	2 (14.3)	0.061
Length of hospital stay, days, median (IQR)	13.5 (10.3–17.0)	7 (5–8)	9.0 (6.5–11.5)	<0.001

*Note*: Post‐ERCP pancreatitis was assessed only in the ETGBD group because it is specific to procedures involving ERCP. Clinical success and recurrent cholecystitis were calculated among patients with technically successful drainage (PTGBD, *n* = 26; EUS‐GBD, *n* = 39; ETGBD, *n* = 14). Length of hospital stay was defined as the number of whole days from the day of hospital admission to the day of discharge.

Abbreviations: ERCP, endoscopic retrograde cholangiopancreatography; ETGBD, endoscopic transpapillary gallbladder drainage; EUS‐GBD, endoscopic ultrasound‐guided gallbladder drainage; IQR, interquartile range; PTGBD, percutaneous transhepatic gallbladder drainage. NA, not applicable.

### 3.3 Surgical Outcomes After Drainage

Of the 84 patients, five patients with ETGBD technical failure and four patients with recurrent cholecystitis were excluded from the surgical outcome analysis. Of the five patients with ETGBD technical failure, three subsequently underwent PTGBD and two underwent EUS‐GBD. The four recurrent cases comprised two patients each in the PTGBD and ETGBD groups. Consequently, 75 patients (PTGBD, *n* = 24; EUS‐GBD, *n* = 39; ETGBD, *n* = 12; Figure 1) were included in the final analysis (Table 3).

**TABLE 3:** Surgical outcomes after drainage.

Variable	PTGBD (*n* = 24)	EUS‐GBD (*n* = 39)	ETGBD (*n* = 12)	*p*‐Value
Time from drainage to cholecystectomy (days), median (IQR)	36 (19–63)	57 (45–79)	65 (37–86)	0.041
Initial surgical approach, *n* (%)				0.041
Laparoscopic	21 (87.5)	39 (100)	9 (75.0)	
Open	3 (12.5)	0	3 (25.0)	
Conversion to open surgery	1 (4.2)	1 (2.6)	0	0.740
Operative time (min), median (IQR)	187 (127–235)	153 (126–214)	169 (152–187)	0.321
Estimated blood loss (mL), median (IQR)	20 (10–120)	50 (10–100)	35 (20–70)	0.646
Length of postoperative hospital stay, days, median (IQR)	5 (4–7)	4 (4–6.5)	4 (4–5)	0.216
Postoperative complications, *n* (%)	2 (8.3)	5 (12.8)	2 (16.7)	0.680
Surgical site infection	1 (4.2)	2 (5.1)	1 (8.3)	
Intra‐abdominal abscess	1 (4.2)	2 (5.1)	0	
Pneumonia	0	1 (2.6)	1 (8.3)	

*Note*: Data are presented as median (IQR) or *n* (%). The initial surgical approach denotes the approach selected at the beginning of surgery; conversion to open surgery denotes a subsequent change from an initially laparoscopic procedure to an open procedure.

Abbreviations: ETGBD, endoscopic transpapillary gallbladder drainage; EUS‐GBD, endoscopic ultrasound‐guided gallbladder drainage; IQR, interquartile range; PTGBD, percutaneous transhepatic gallbladder drainage.

The interval from drainage to cholecystectomy differed significantly among the groups (*p* = 0.041), with the shortest interval observed in the PTGBD group. The initial surgical approach also differed significantly (*p* = 0.041): all patients in the EUS‐GBD group underwent an initial laparoscopic approach, compared with 87.5% in the PTGBD group and 75.0% in the ETGBD group. Conversion to open surgery, operative time, estimated blood loss, length of postoperative hospital stay, and overall postoperative complication rates did not differ significantly. Postoperative complications were infrequent and comprised surgical site infection, intra‐abdominal abscess, and pneumonia.

## 4 Discussion

In this retrospective, single‐center study, we compared PTGBD, EUS‐GBD, and ETGBD as BTS strategies for AC and found that technical and clinical success were generally consistent with prior reports. Technical success rates were 100% for PTGBD and EUS‐GBD but 73.7% for ETGBD (*p* < 0.001), with no significant difference in adverse‐event rates across all three modalities [13]. The lower ETGBD success rate likely reflects reliance on cystic duct access, which may be technically difficult in the presence of inflammation‐related stricture, obstruction, or impacted stones [8].

Although clinical success did not differ significantly across the groups, EUS‐GBD achieved clinical success in all 39 patients, consistent with prior evidence of non‐inferiority to PTGBD [14] and, in some settings, higher success than ETGBD [15]. Unlike transpapillary access, EUS‐GBD does not depend on cystic duct patency and can therefore provide effective internal drainage even when transpapillary access is technically difficult.

Although the overall incidence of adverse events did not differ significantly among the groups, the types differed. Drain dislodgement occurred only in the PTGBD group, reflecting a known limitation of external drainage. Tube‐related complications such as dislodgement and occlusion are well‐recognized problems with PTGBD and may require redrainage or additional intervention [16]. By contrast, adverse events in the endoscopic groups were procedure‐related. Peritonitis occurred in 15.4% of patients in the EUS‐GBD group; however, all cases were mild and resolved with conservative management, without prolonged hospitalization. Post‐ERCP pancreatitis occurred in 10.5% of patients in the ETGBD group, consistent with previous results [8], and all cases were mild. These findings suggest that the observed procedure‐related adverse events were manageable with conservative treatment in this cohort.

Recurrent AC occurred in 7.7% of the PTGBD group, which was lower than the previously reported rate of 20% [17]. The recurrence rate in the ETGBD group was similar to that reported previously [15]. By contrast, no recurrence was observed in the EUS‐GBD group in this cohort, which suggests durable internal drainage, although the limited sample size warrants cautious interpretation. Postoperative complication rates did not differ significantly, suggesting that subsequent cholecystectomy was feasible after each drainage method in patients who successfully proceeded to surgery.

The interval from drainage to cholecystectomy differed across the three groups, being the shortest in the PTGBD group (median, 36 days) and longest in the ETGBD group (median, 65 days), consistent with previous results [18]. This difference may partly reflect the nature of PTGBD as an external drainage procedure, in contrast to EUS‐GBD and ETGBD, which provide internal drainage without external tube management. However, no formal prospective BTS‐management protocol was used during the study period, and surgical timing was therefore determined individually according to the patient's condition and surgeon judgment. Consequently, residual confounding by unmeasured factors, including case complexity, residual inflammation, surgeon preference, and anatomical factors, cannot be excluded, and the effect of the drainage‐to‐surgery interval on surgical outcomes could not be determined. Notably, the ETGBD group had the longest drainage‐to‐surgery interval yet the lowest rate of initial laparoscopic approach (75.0%, vs. 100% for EUS‐GBD and 87.5% for PTGBD), contradicting the hypothesis that a longer waiting interval alone increased the laparoscopic approach rate. Therefore, the observed intergroup differences are more likely to reflect residual confounding than a causal effect of the drainage‐to‐surgery interval itself.

In the EUS‐GBD group, a fistula between the gallbladder and gastrointestinal tract is a characteristic intraoperative finding. The fistula generally matures 4 weeks after EUS‐GBD [19]. Although increased inflammation or adhesions may increase surgical difficulty, no substantial difficulty in fistula management or apparent adverse association with subsequent cholecystectomy was observed in this cohort.

All EUS‐GBD procedures in this study were performed using DPPS rather than lumen‐apposing metal stents (LAMSs). Although LAMSs provide better sealing of the puncture tract and may reduce bile leakage [20], they may also induce stronger tissue reactions and increase surgical difficulty during subsequent cholecystectomy [21]. In Japan, LAMSs were approved in June 2025 and are currently used mainly in patients who are not surgical candidates; therefore, their use in BTS cases is generally off‐label. Additionally, most of the study period preceded their introduction, and LAMSs were therefore unavailable for most patients in this cohort. Our results therefore specifically reflect EUS‐GBD with DPPS in the BTS setting. Although DPPSs have been associated with bile leakage and pneumoperitoneum during the procedure [22], these events were not associated with apparent adverse effects on subsequent cholecystectomy in our cohort. Technical refinements, such as maintaining the inner sheath in a halfway back position during flushing, may further reduce leakage of infected bile before fistula formation and help avoid conversion to open surgery [23].

Previous studies comparing surgical outcomes after drainage have shown no significant differences between PTGBD and EUS‐GBD in adverse event or conversion rates, although operative time was shorter with EUS‐GBD [24]. Another study showed no significant difference in adverse‐event rates between PTGBD and ETGBD, but ETGBD was associated with lower conversion rates and shorter operative times [18]. In the present study, although BMI differed significantly among the groups, conversion to open surgery, operative time, estimated blood loss, postoperative hospital stay, and postoperative complication rates did not differ significantly. Our findings suggest that all three drainage modalities enabled subsequent cholecystectomy with acceptable surgical outcomes when BTS management was achieved.

This study has some limitations. First, the relatively small, retrospective, single‐center design, together with non‐randomized, physician‐determined treatment allocation, may have introduced selection bias and residual confounding affecting both drainage and surgical outcomes. Second, surgical outcomes were analyzed only in patients who proceeded to cholecystectomy, precluding an intention‐to‐treat comparison across the three strategies. Third, the limited number of secondary‐endpoint events may have left some comparisons underpowered. Finally, the extended study period may have introduced heterogeneity in devices, operator experience, and clinical care pathways. Larger multicenter studies with multivariable adjustment or propensity score‐based analyses are warranted to clarify the optimal drainage strategy.

In conclusion, we demonstrated that PTGBD, EUS‐GBD, and ETGBD are feasible BTS strategies for acute cholecystitis. EUS‐GBD achieved high technical and clinical success with acceptable safety and was not associated with apparent adverse effects on subsequent cholecystectomy in this cohort. However, owing to the retrospective design and potential selection bias, these findings should be interpreted with caution. The drainage strategy should be individualized according to patient condition, anatomical feasibility, and clinical circumstances.

## Author Contributions

**Conceptualization**: Keiki Nagai; **investigation**: Keiki Nagai, Hiroki Uechi, Yuji Koyama, Shintaro Tsujikawa, Eisuke Suzuki, Seitaro Tsujino, Ken Ishii, and Atsuki Nagao; **writing – original draft preparation**: K.N.; **writing – review and editing**: Yuji Fujita and Masato Yoneda; **supervision**: Yuji Fujita and Masato Yoneda. All authors have read and agreed to the final version of the manuscript.

## Funding

The authors have nothing to report.

## Ethics Statement

The study protocol was approved by the Institutional Review Board of NTT Medical Center Tokyo (Approval No. 200037461‐01).

## Consent

The requirement for written informed consent was waived because of the retrospective study design, and an opt‐out approach was used for participant notification.

## Conflicts of Interest

The authors declare no conflicts of interest.

## Data Availability

The data that support the findings of this study are available from the corresponding author upon reasonable request.
